# Augmented Reality in Maintenance—History and Perspectives

**DOI:** 10.3390/jimaging9070142

**Published:** 2023-07-10

**Authors:** Ana Malta, Torres Farinha, Mateus Mendes

**Affiliations:** 1 Coimbra Institute of Engineering, Rua Pedro Nunes-Quinta da Nora, Polytechnic Institute of Coimbra, 3030-199 Coimbra, Portugal; 2 RCM2+ Research Centre for Asset Management and Systems Engineering, ISEC/IPC, Rua Pedro Nunes, 3030-199 Coimbra, Portugal

**Keywords:** augmented reality, maintenance, task assistance

## Abstract

Augmented Reality (AR) is a technology that allows virtual elements to be superimposed over images of real contexts, whether these are text elements, graphics, or other types of objects. Smart AR glasses are increasingly optimized, and modern ones have features such as Global Positioning System (GPS), a microphone, and gesture recognition, among others. These devices allow users to have their hands free to perform tasks while they receive instructions in real time through the glasses. This allows maintenance professionals to carry out interventions more efficiently and in a shorter time than would be necessary without the support of this technology. In the present work, a timeline of important achievements is established, including important findings in object recognition, real-time operation. and integration of technologies for shop floor use. Perspectives on future research and related recommendations are proposed as well.

## 1. Introduction

This is a review article that aims to summarize the work carried out in the area of augmented reality to help with maintenance tasks using deep learning neural networks in object detection. We intend this review to help readers understand this area and its immense potential for future projects.

The evolution of technology has been used in supporting, solving, and optimizing various problems in different contexts of daily life. Augmented Reality (AR) is one such technology, allowing virtual elements such as objects, texts, graphics and other types of data to be superimposed onto images of real environments.

This technology can be found in the context of games, such as the popular POKEMON GO [1], and camera filters that superimpose effects onto the original view. In its simplest form, AR can be achieved with simple equipment that is available for most citizens of the world, requiring only a mobile device with a camera and reasonable processing capacity [2].

AR can be applied in open or closed spaces using adequate hardware, and has many different uses in education, healthcare, and more. Previous literature reviews on the use of augmented reality in other areas have been presented in [3,4,5].

Achieving AR with a simple cell phone is often the cheapest and most practical approach [6]. However, devices such HMD (Head Mount Display) can provide a superior experience. Users can have their hands free to perform different tasks. Thus, studies and technological advances are beginning to emerge showing that HMDs can help professionals in different areas, including medicine, teaching, and maintenance. Such devices are considered “wearable”, allowing users to see real and virtual information at the same time. Most have various types of connectivity, GPS receiver, microphone, voice recognition, and even gesture recognition [7].

In general, augmented reality contributes to standardization as well as to friendlier and more efficient workflows through the use of contextualized and personalized information [8]. In areas such as maintenance, where all processes of learning, training, and communication imply investment, decreasing the down time of equipment can help to provide greater returns on direct financial investments. In this context, AR can emphasize the importance of the preceding investments, including the value that added due to shorter deployment and training times.

Currently, maintenance protocols require printed or digital manuals that explain maintenance procedures in detail, and these require careful training and study. Despite progress in human–machine interaction, newer and more complex equipment may contain digital systems that are highly complex and which require demanding and careful maintenance.

If maintenance technicians are able to use augmented reality glasses to help with maintenance tasks, this can reduce costs, increase production capacity, reduce safety risks, and optimize the learning process, among other advantages [9]. One important advantage is that assistance with maintenance tasks in which the workflow is directed from AR glasses worn by technicians can help to ensure that no procedures are missed. Furthermore AR assistance can reduce the need for oversight by more experienced personnel during the training processes.

Thus, AR is an important step towards the digitization of services taking place all over the world. On the other hand, it poses new challenges for energy supply and demand, as AR headsets and similar devices require large processing and communication capacities in real time, which draws considerable amounts of energy from portable batteries. Nonetheless, AR in maintenance is an area of active research for training and guidance of maintenance technicians, and interest is growing.

Augmented reality initially used markers; however, the focus of this study is on the potential of integrating AR with deep learning algorithms in object detention, avoiding the use of markers.

Modern deep learning algorithms have played a key role in the development of AR. For the first time in history, convolutional neural networks are capable of automatically recognizing objects in images with very good accuracy and precision. This capacity makes it possible to identify objects in the user’s field of view and take actions based on what is visible, as well as to assess the results of many actions that change the environment. Therefore, deep learning methods are taking the AR experience to a whole new level, expanding its areas of application while opening new lines of applied research.

The present research identifies important steps in the evolution of augmented reality and provides a comparative analysis of several studies on the use of augmented reality in maintenance, including automotive, military, and other use cases. Important algorithms, methods, ideas, and trends are identified. In order to optimize this process, this review further aims to show how the use of deep neural networks can be combined with augmented reality to identify and locate objects automatically, and how this can be explored for better application of AR technologies.

Section 2 describes the methodology followed during the review article. Section 3 contains an introduction to the concept of augmented reality and its historical evolution. Section 4 shows examples of the application of augmented reality for maintenance tasks. Section 5 introduces computer vision technology and neural networks with augmented reality in the maintenance context. Section 7 presents a discussion of future research perspectives. Finally, the last Section 8 provides a brief conclusion.

## 2. Research Methodology

In the present work, we have conducted a systematic literature review (SLR) on augmented reality, focusing mostly on landmark developments and industrial applications.

In the first phase, the two types of augmented reality are considered, namely, approaches with and without markers. A search was carried out for articles related to this topic through the “Google Scholar” platform using the following main keywords: “Augmented Reality”; “Maintenance”; and “Artificial Neural Networks”. The resulting articles were analyzed and compared in a number of aspects. This was a preliminary investigation, and was smaller than the full review, as it was only introductory in nature compared to the main focus on studies in which augmented reality techniques were used for maintenance tasks.

In the second phase, because the research on neural networks is quite extensive, only those approaches deemed most relevant were reviewed in detail. A comparison of several studies involving different architectures for deep learning neural networks was conducted. In order to filter the articles, we decided to focus on articles published after 2019 up to the present. In specific cases, such as the use of AR in electric power systems, it was necessary to move the search date back to 2017 in order to find relevant studies, as described in Section 4.3.

The main sources we used were Web of Science, IEEE Xplore, and ScienceDirect.

## 3. Augmented Reality

### 3.1. Basic Techniques for AR

Augmented reality can be used in several ways. Estrada et al. evaluated several of these techniques in a project for an AR application in engineering lab training [10]:

**Marker-based AR:** This technique requires the use of visual markers such as QR codes or other patterns, which represent reference points for the virtual object. When the AR system recognizes these markers, for example through a camera, the virtual objects or information are superimposed on the real view.

**Markerless AR:** Unlike the previous technique, these systems do not require specific markers. Instead, they use computer vision techniques such as localization or object recognition to detect the environment that the user is in and to place virtual objects in the user’s view.

**Location-based AR:** This technique collects real-time information through the use of GPS or other device location systems to overlay AR content based on the user’s geographic position.

**Projection-based AR:** Here, virtual content is applied to real-world objects or physical surfaces via projectors or projector-equipped glasses that project the virtual content, avoiding the need for screens or other equipment.

**Recognition-based AR:** A technique that involves recognizing real-world objects or images and then overlaying relevant information or virtual content onto the identified objects.

**Outlining-based AR:** This type of AR uses image recognition to obtain a better understanding of the current environment; users can create contours or shapes and highlight components of the real world using special cameras.

**Motion tracking:** Motion tracking techniques allow AR systems to follow the user’s movements, and as such are able to adjust the position and/or orientation of virtual objects depending on the user’s movements. This allows for greater interaction with AR content, providing a more immersive and dynamic experience.

**Simulated Physics and Interactions:** In this approach, virtual objects can interact with the real-world environment or with each other in order to enhance the realism of AR experiences.

**Figure 1:**
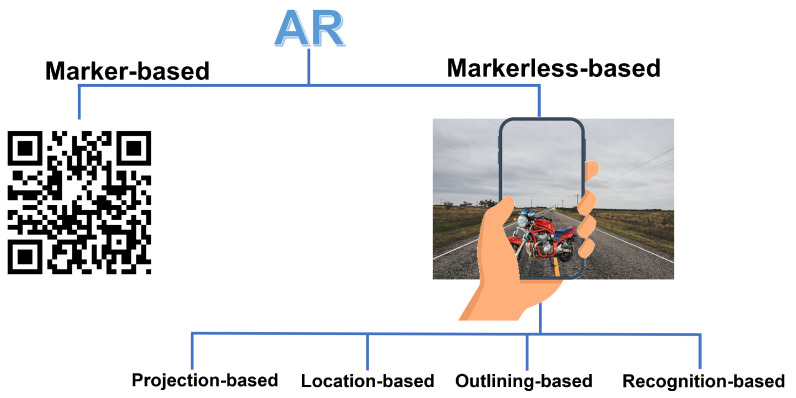
Basic techniques commonly used in AR.

Figure 1 shows the two major divisions of AR, one using markers and the other without markers, and presents examples of the techniques described above.

### 3.2. Virtual Objects

Along with the concept of AR, there are other terms that refer to the use of virtual objects. Virtual Reality (VR) and Mixed Reality (MR) are often confused with augmented reality. VR is a technology that allows users to experience a virtual environment through VR glasses. MR refers to the inclusion of virtual objects in a real environment or the inclusion of real elements in a virtual environment.

There are different types of augmented reality, and each may be better suited for particular uses, although they all share common characteristics. Marker-based AR applications use target images (markers) to position objects in a given space. These markers determine where the application places the 3D digital content in the user’s field of view (FOV) [11]. Markerless AR analyzes the real environment and places digital elements on a recognizable feature, such as a flat surface; thus, instead of being attached to a marker, digital elements are placed based on geometry [12].

There are several techniques for presenting virtual data. One technique is based on the use of portable screens, for instance, mobile devices such as smartphones or tablets, providing users with a video view of the physical environment that is then amplified by corresponding virtual content. Another technique is a head-mounted visor which is worn on the user’s head, possibly as part of a helmet. These devices use optical visualization methods that allow users to view the real world environment with their eyes and see virtual content overlaid by holographic optical elements [13].

One of the simplest ways of producing virtual or augmented reality effects is to use projection devices, sometimes called spatial displays. These devices directly display virtual content on the surfaces of real objects, and can be naturally enlarged to permit collaboration between a group of people [14,15].

In general, all types of displays have advantages and disadvantages. For handheld devices, the main disadvantages are that they constrain the use of the hands and that the information, is restricted to the size of the screen. On the other hand, the ability to easily take these devices anywhere represents a major advantage. Conversely, a form of spatial display that interconnects a camera and a projection device allows hands-free use and can maximize user immersion [6]. However, it is not as easy to transport these devices. HMDs ultimately combine both advantages, allowing hands-free use and being easily transported. The main disadvantage of these devices is that during prolonged use there may be discomfort for the user, although technology has been developed to make these devices increasingly comfortable for prolonged use.

Smart glasses are a type of intelligent augmented reality device that offer various opportunities in work environments [16]. They interact with the physical world and the user in real time. Other types of devices, such as smart hats or helmets, may be a better option if the operator wears graded glasses [17]. In applications that use AR glasses, it has been determined that these devices can help employees with various processes and operations, increase the speed and quality of work, provide a safer working environment, and aid in communication between workers and machines [18].

Industry 4.0 proposes the use of predictive technologies for tasks such as manufacturing and maintenance in the industry of the future, with connected machines as part of a collaborative community. This evolution requires the use of advanced predictive tools to ensure that data can be systematically processed in order to extract information that can explain uncertainties and lead to more informed decisions [19,20].

### 3.3. History of Augmented Reality

The history of augmented reality started around the year 1957, when Morton Heilig invented the Sensorama, which delivered visuals, sounds, vibrations, and smells to the viewer. Despite not being controlled by a computer, this was considered the first example of an augmented reality experience [21]. In 1968, Ivan Sutherland, an electronics engineer at Harvard University, created the first HMD, The Sword of Damocles. This device was able to convey visual effects, sounds, and smells to the user [8]. As computers at the time were quite limited, it was only possible to see wireframes in real time.

The term AR officially emerged in the year 1990 when it was coined by Thomas P. Caudell, from Boeing. Caudell created a system [22] based on augmented reality to assist mechanics working for Boeing. After putting on special glasses, users were assisted by the tool in finding the correct connections of cables and wires in aircraft engines. This made life a lot easier for mechanics, who could save time by not having to read huge airplane manuals.

In 1992, Louis Rosenberg developed the first successful fully immersive AR system for the US Air Force [23]. Virtual fixtures allowed the military to control virtually guided machines to perform operational tasks in space.

In 1993, the KARMA (Knowledge-based Augmented Reality for Maintenance Assistance) project was the first project conducted for maintenance applications using a see-through head mounted display to display instructions about how to perform a task on a printer [24].

In 1996 Schmalstieg et al. developed Studierstube, the first collaborative AR system. With this system, multiple users could experience virtual objects in the same shared space. Each user had a tracked HMD and could see correct-perspective stereoscopic images from an individual viewpoint [25].

In 1997, Feiner et al. developed the first outdoor AR system at Columbia University. This machine used an HMD with GPS and orientation tracking.

The history of markers began in 1998. Jun Rekimoto [26] introduces a new method to build an augmented reality system using printed 2D matrix codes applied to a video image [27].

In 1999, this technology began to be applied in industrial contexts. NASA (National Aeronautics and Space Administration) created a hybrid synthetic vision system of their X-38 spacecraft. In the same year, the first work about ARToolKit, the most popular open-source library for AR, was published [24].

In 2001, an AR interactive maintenance guide was developed to replace paper-based instructions, and in 2004 Stuart Gooseet al. [28] created an AR system using voice control for the inspection of a water distribution system.

In 2007, several examples of systems integrating AR with CAD (Computer-Aided Design) models appeared [15,29].

In 2008, Schönfelder and Schmalstieg [30] proposed a Planar-based system for an AR display on wheels with external tracking which provided fully interactive real-time discrepancy checking for industrial facilities.

**Figure 2:**
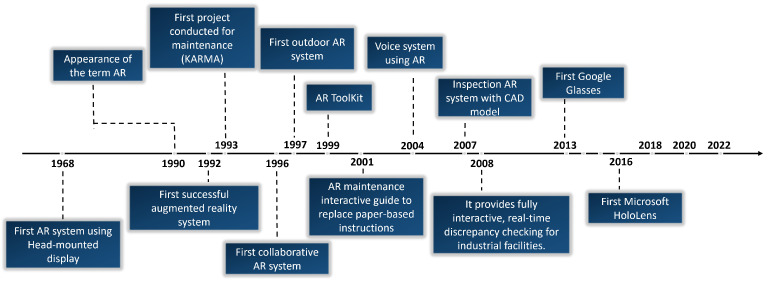
Timeline of developments in Augmented Reality, highlighting the most important advances for industrial applications.

The first Google Glasses model appeared in 2013, followed by the Microsoft HoloLens in 2016. Both brands have subsequently developed new models [31,32]. Figure 2 presents a summary of the main advancements in the field of AR. As the figure shows, the most remarkable developments took place before 2016. The area remains very active, however, as technologies mature and AR becomes more prevalent.

## 4. Augmented Reality in Maintenance

### 4.1. Automobile Industry Applications

Several companies in the automotive sector have been developing AR applications to permit users to interact with their vehicles. As early as 2010, Volkswagen presented an Augmented Reality version of the Golf model on its website, and in 2012 Volvo provided the opportunity to see the inside of a model, the Volvo V40, using 2D markers to transform 3D images through an augmented reality experience [33]. The Hyundai Motor Company developed an application to permit that users to access information about their car through their smartphone or tablet [33,34].

BMW has developed augmented reality techniques to support maintenance work for complex technical innovations and vehicles service. This solution for AR-based repair guidance consists of a markerless CAD-based model from BMW that uses a virtual camera with a 90${}^{\circ }$ field of view; the length of each video sequence is 100 frames, with a resolution of 640 × 480 pixels. This system is able to deal with different illumination conditions during the tracking stage and to automatically recover from occasional tracking failures. Two hardware solutions were experimented with based on a wireless mobile setup: a monocular full-color see-through video HMD and a monochrome optical see-through HMD [35]. The system proved to be useful for different maintenance and repair scenarios [36].

Boboc et al. have produced a systematic literature review on AR in the automotive industry [37]

### 4.2. Military Applications

AR has been widely studied over the years for military applications, where it has found uses in control, support, and especially equipment maintenance. The ARMAR (Augmented Reality for Maintenance And Repair) project [38] created by the US Air Force in conjunction with Columbia University aimed to evaluate the effects of AR on equipment maintenance and related personnel training.

Henderson et al. [20] analyzed the implementation and testing of an augmented reality system to support United States Marine Corps (USMC) mechanics performing routine maintenance tasks inside the turret of the LAV-25A1 armored vehicle. Because they did not have access to the vehicle, they used an extended set of 3D laser scans to create a mostly virtual mock-up of the turret, which was used in the lab during development. The first pilot test involved prototype testing with Marine Corps Logistics users. They used this pilot test to refine the tracking configuration and gather feedback from users on the interface and techniques. A second pilot test involved four mechanics from the population recruited for a practical user study.

They experimented with two HWD (Head-Worn Device) screens. These displays are more recently referred to as head-worn displays, but historically have been referred to as head-mounted displays (HMD) [39]. In the pilot test, they found that vehicle assemblies directly in front of and behind the seats prevented users from freely moving their heads while using the relatively large HWD nVisor. This showed that a custom HWD with an 800 × 600 resolution headplay with a 34${}^{\circ }$ diagonal field of view would be better.

The study was intended to compare the prototype with the use of user manuals. During each task, the LCD (Liquid Crystal Display) presented a single static 3D scene rendered in VR. Each static scene was presented using the same engine used to generate the virtual content for the AR condition, and described identical text instructions, 3D labels, close-up graphics, and animated sequences. Additional 3D models were added to the scene to represent the central component of interest and important surrounding context. To control the overall effects of using the HWD, a third condition was added which included an unmonitored version of the AR prototype. This Head-Up Display (HUD) condition used fixed graphics on the screen to present text instructions and close-up views identical to those in the AR condition. When experiencing the HUD condition, participants used the same HWD as in the AR condition to interact with the application using the same pulse controller as in the AR and LCD conditions.

The authors selected eighteen tasks (e.g., replacing a pump) performed as part of a larger maintenance sequence.

The average task completion times for each condition were 42 s (AR), 55.2 s (HUD), and 34.5 s (LCD). The mean task location times were 4.9 s (AR), 11.1 s (HUD), and 9.2 s (LCD).

The authors stated that their qualitative results provide additional incentives for the application of AR in maintenance tasks. They further noted that future AR systems should contain lighter and more comfortable screens with larger viewing areas and higher resolutions.

LaViola et al. [40] presented a prototype of the ARWILD system, examining the fusion of intelligent tutoring with augmented reality for use in hands-on immersive training for psychomotor tasks. The noted future plans to extend the system to include more complex training scenarios, such as threat assessment and mission rehearsal monitored by a GIFT-based tutor.

### 4.3. Electrical Energy Systems Applications

Zhukovskiy et al. [41] and Koteleva et al. [42] developed augmented reality modules for performing electrical equipment maintenance. In both cases, the authors concluded that the use of the augmented reality system can reduce the cost of maintaining electrical equipment. The development of a maintenance script that describes various tasks can help to reduce errors and increase the quality of maintenance interventions.

In another study [43], an AR system for maintenance and repair of an electrical distribution system was proposed. A program called Vuforia was used for object recognition of 3D models of objects designed in CAD within the AR application. The systen uses two databases; one is used if connectivity is not available, in which case the system produces data and stores it in the device database. When connectivity is available, the system is designed to allow data stored on the device to be transmitted to the central database. Tests succeeded in reducing costs on several levels by minimizing travel and the resulting time expenditure. In addition, the level of technician safety can be increased and the risk of human error in the repair and maintenance process reduced.

In 2017, a number of studies were conducted; Peng et al. [44] used AR technology in the power sector to improve operations and aid in training the operations staff of the substation power grid. This intelligent training system was able to increase the effectiveness of training and promote the use of emerging technologies. The French electricity company “Enedis” has implemented an application to facilitate the use of low voltage control panels and medium voltage devices in order to reduce possible errors through a series of AR instructions. This solution incorporates an application that allows field personnel to easily locate faults in underground cables using smart glasses. The potential advantages of this solution include reduced intervention preparation time, increased operator safety level, improved comfort, and reduced time on location [45,46].

### 4.4. Other Areas of Application

Henderson et al. [47] proposed a test study that used augmented reality for the process of assembling and disassembling a combustion chamber. In their setup, dynamic 3D arrow is presented to the user through a pair of AR glasses. Initially, a large red arrow indicating the direction and magnitude of movement is used to align the can and cone. As alignment grows nearer, the arrow decreases in size and changes color, eventually disappearing when alignment is complete.

The prototype used an HWD with a diagonal field of view of 60${}^{\circ }$ per eye, 40% optical transmissivity, and an image with a resolution of 1280 × 1024 in each eye.

The study was conducted by viewing information through th glasses and comparing the results to viewing the same information on an LCD. The object location time was slightly longer with the glasses; however, the task execution time was 21.31 s faster (46.8%) using AR. The authors stated that the localization time was faster using the LCD because because the participants easily knew where the object was located. The average difference between the ideal orientation and that achieved by the user was 0.08 radians for the AR and 0.36 radians for the LCD. The average accuracy rate in the AR condition was 95.3 percent, compared to 61.7 percent in the LCD condition.

Moutriz et al. [48] developed a framework for supporting real-time remote maintenance based on AR. Their first step was to develop the software for the cloud platform. They developed architecture of the framework to support a variety of platforms, including PCs, HMDs such as Microsoft HoloLens, and handheld devices. The applicability of the proposed framework was tested and validated in a lab-based machine shop as well as in a real industrial setting.

The results indicated that the functional requirements discussed during the problem modeling phase research work were successfully addressed and that the resulting zero-time AR content creation tool further minimized the MTTR (mean time to repair).

Tang et al. [49] conducted a study to evaluate the use of HMDs for presenting AR-based instructions. Their study was conducted with 75 participants using LEGO Duplo blocks. The participants had to complete assembly tasks following instructions presented through (1) printed media, (2) instructions on a monitor, (3) instructions on an HMD, and (4) spatially recorded AR instructions on an HMD. This study showed AR-based systems can improve task performance in terms of mean time and number of errors compared to other media, while reducing the mental workload required for assembly tasks [50].

### 4.5. Use of CMMS

Ariasyah et al. [51] discussed the integration of AR systems through a CMMS (Computerized Maintenance Management System). These systems aim to plan preventive and predictive actions in order to minimize breakdowns and unexpected delays. Using a CMMS, it is possible to gather information about the equipment, maintenance data and scheduling, failure history, and parts inventory.

The development of an AR-CMMS system for maintenance was demonstrated through the example of a 3D printer. In this study, two types of failures were considered: an easy “level” failure and a more difficult one. Maintenance instructions can be presented through HoloLens glasses in text format or verbal messages followed by labels that identify the target location and animated 3D models or video clearly showing how the task should be performed. In this study, a marker was used to identifies the object of interest.

The authors stated that the AR-CMMS system allows unexpected breakdowns to be dealt with immediately by the operator.

Aschauer et al. [52] presents an implementation of an open-source remote AR support application based on free software components. The framework connects a portable device from an on-site worker to a remote expert using a desktop application. The purpose of the evaluation was to obtain information about the potential of the prototype in direct comparison with the paper maintenance instructions. Both groups took almost the same amount of time to perform the maintenance task, at 4 min 55 s for the paper instructions and 4 min 57 s with the AR remote support tool. However, the situation was different when looking at the overall error rate; 53% of PC maintenance tasks were performed incorrectly using paper instructions, while only 13% of the tasks were performed incorrectly with the use of remote AR support.

### 4.6. Discussion

The results of the studies detailed above and summarized in Table 1 present the relevant problems, some of the hardware and software solutions used to date, and the authors’ main conclusions. In all of the cited studies, the results were satisfactory and demonstrated the immense capabilities that these systems can provide in diverse industries and fields, including automotive, military, educational, industrial, and many more.

It is important to realize that there are several studies that, although less detailed, demonstrate the potential applicability of augmented reality for teachers, educators, and trainers, smart mobile terminals, and even in the area of medicine, with a focus on visualization for surgical support [53,54]. Overall, it can be concluded that although there may be limitations in terms of data or available technology, augmented reality is slowly progressing and increasingly showing more areas of application.

## 5. Applications of Machine Learning for AR

**Figure 3:**
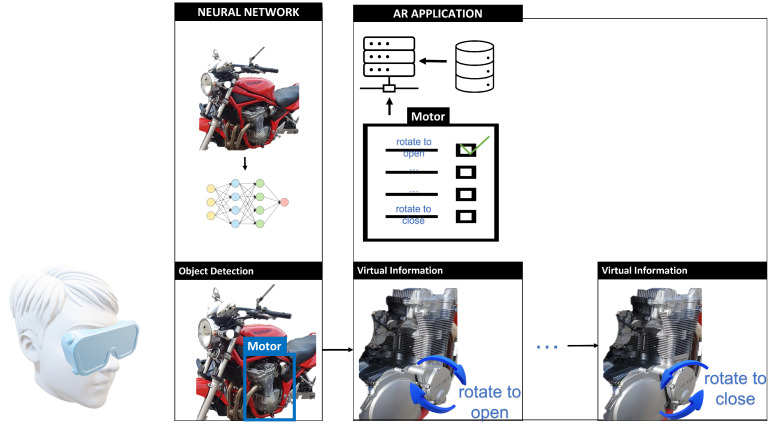
Pipeline of an AR application with a neural network used for object detection.

Modern machine learning algorithms are playing a key role in the development of new AR applications. The use of deep learning techniques can help to overcome several typical shortcomings of marker-based and markerless AR. Through deep learning, it is possible to obtain faster and more accurate detection [55]. Figure 3 shows the pipeline of an AR application with a neural network used for object detection. After the artificial intelligence system uses the neural network for object detection, the AR system receives the relevant information from the database about the object detected by the user. An example of such a system is a CMMS that contains work orders and maintenance procedures. As the user completes a procedure, the next one is presented in the form of virtual information.

This section reviews a number of the latest applications.

### 5.1. Object Recognition and Tracking

To automatically trigger certain actions in an AR scene, it is necessary to detect related events, which ideally takes place automatically. Different types of events can be used, such as sounds or objects in the scene. The presence of objects in the field of view is one of the preferred methods. In the past, one of the most used solutions to overcome this problem was the use of fiducials, or artificial markers placed in the scene [56]. The markers can be anything unique or rare, but should be simple (e.g., a QR code) and must be in the right location to appear in the camera’s field of view at the right time [57].

Untagged augmented reality, known as location-based reality, provides location-based data that can be provided via sensors such as digital compasses, accelerometers, a speed meters, or GPS [57]. Marker-based AR is often less complex, and allows for more precise solutions.

As the use of artificial markers represents a major constraint on AR usage in industrial environments, target objects must be found based on their natural characteristics [24]. To this end, it is possible to use the power of artificial neural networks for the recognition of objects, machines, or any other components. The first results were obtained using computational vision (CV) algorithms; however, modern CNN networks are much more accurate and easier to implement than older CV techniques.

CV has changed traditional construction management by enabling automatic activity recognition, object tracking, and performance monitoring [58]. Advances in these techniques have made them extremely suitable for AR applications, as they can use images or video captured by an AR device’s built-in camera to provide functions such as visual tracking and image registration [13].

In industry, computer vision is used as a tool to support quality control, object counting, identification of worn equipment, and more [59,60]. In routine inspections, real-time defect recognition and feedback to inspectors is made possible by simplified image capture devices [61].

Ahmadyan et al. [62] proposed an application of AR for virtual shoe fitting. In their approach, a detection network makes predictions with an average accuracy of 0.59 and the model output includes shape information, for instance, a segmentation mask. Using a Samsung S20 mobile phone, the object detector runs at 26.5 fps on the GPU, while the 3D tracking runs at 30 fps on the CPU.

### 5.2. Image Feature Matching and Pose Estimation

Recent techniques based on deep learning are used in feature matching as well. This technique can be combined with AR, and involves identifying and tracking of points of interest in an image captured by a camera. Accurate matching of features between real-world images and virtual images is essential to ensure accurate alignment of virtual objects. Sarlin et al. [63] proposed a system called “LaMAR” to compare and evaluate different techniques and algorithms used for localization and mapping in AR.

Algorithms based on convolutional neural networks and recurrent neural networks have been used in accurate real-time pose estimation. This method of recognition in the AR world is essential, as it allows virtual objects to be correctly superimposed on the real environment. Konstantinidis et al. [64] presented the “MARMA” project, which uses pose estimation methods to track and identify relevant components and objects for repair procedures in the context of Augmented Reality (AR).

### 5.3. Models Using Deep Learning

Deep neural networks are very good at object detection, and their application is changing the way AR models operate. Several key works are reviewed in this section. Because of their importance for real-time operation, applications that use YOLO and MobileNet are reviewed separately.

#### 5.3.1. Models That Use CNN

Mourtzis et al. [65] presented a framework to generate instructions with AR technology based on Convolutional Neural Networks (CNN). This architecture contains three modules: (1) the spatial recognition module responsible for analyzing the machine tool and, through the CNN, recognizing the components that compose it; (2) the CNN module responsible for the spatial recognition of the technician’s physical environment; and (3) the AR instruction generation module responsible for generating the AR instructions.

For development, the Microsoft HoloLens was used with a desktop PC. For the AR module, the Unity 3D game engine was used together with the MRTK library and Vuforia API. This API was used to enable the generation and viewing of AR scenes.

To begin the procedure, the video is first processed by the CNN, which recognizes the equipment. After completing this step, the technician validates the recognition result, then communication to the third module is performed, which generates the AR instructions to be transmitted to the glasses that become visible to the technician. In the various tests performed, all technicians were able to perform the tasks; although in certain cases they were not confident due to lack of knowledge and experience, all followed the instructions and managed to complete the tasks. Regarding the process time, the average time for disassembly was 60 min, including familiarization with the structure. The average time for an experienced technician was around 25 min. The authors concluded that in order to successfully develop the proposed architecture it was necessary to a computing cluster, optimize the CNN architecture, and perform new experiments using the same framework within a real-world context.

Zhao et al. [66] proposed a similar client/server architecture for a maintenance system using the Microsoft Hololens as the hardware platform along with augmented reality and recognition via artificial intelligence. Their proposed architecture was divided into three subsystems: (1) an artificial intelligence training subsystem to train the dataset; (2) the resource management subsystem for managing users, models, maintenance steps, maintenance tools, audio presentation, etc.; and (3) the AR subsystem, including process control, target detection, target recognition, resource assembly, and the interactive augmented reality display.

The artificial intelligence training subsystem was divided into client-side and server-side training. On the client side, the training model is used to directly perform recognition operations, greatly reducing the real-time calculation pressure and making the system faster and more responsive.

This paper proposed and discussed a remote control tower maintenance and induction system. An algorithm was trained and a recognition model was generated. Resources such as description, fault type, and troubleshooting method were managed uniformly and exported to the resource management system, then received by the AR system, which used Microsoft’s HoloLens augmented reality glasses.

In the proposed system, the intended functions are explained and displayed in the form of graphical and 3D models, description of steps, warning information, etc. Maintenance jobs can replay, pause, and rewind the installation, debugging, and disassembly steps.

The authors concluded that the system improved maintenance efficiency and significantly improved the training process with respect to complex equipment, which is of great importance in improving the information level and combat capability of the military.

Lai et al. [67] presented the development of an AR-based mechanical assembly instruction system aimed at improving worker performance. A tool detector was developed using a Faster R-CNN model trained on a CAD synthetic tool dataset, and was able to detect real physical tools with an average precision of 84.7%. Experiments with this system on a spindle motor assembly showed that the system reduced the assembly completion time and number of errors by 33.2% and 32.4%, respectively.

Zheng et al. [68] developed a framework to create and manage the aircraft cable assembly process based on a wearable AR device and text recognition using a CNN.

Park et al. [69] proposed a new mobile AR based on deep learning to allow 3D spatial mapping between virtual and real objects. Their proposed approach can augment a virtual model to suit the 3D position and pose of a real object. They used a Mask R-CNN based on instance targeting results. The proposed method extracts 3D point cloud data corresponding to each real object from snapshot-based 3D point cloud data, then performs matching between the virtual model and the real detected object. The virtual model is spatially mapped to the real object using the 3D position and pose of the real object. To prove the applicability of the proposal, applications such as task assistance for repair and assembly were implemented.

Wang et al. [70] presented a two-stage knowledge-based deep learning algorithm enabling automated damage detection in real time using AR smart glasses.

Coscetti et al. [71] described a software prototype realized to perform image recognition for the visualization of scenes in an augmented reality system to support maintenance and control of the transformation line of a tissue-converting factory through an artificial intelligence module that provides suggestions and instructions to the operators.

Sun et al. [72] presented an AR system that integrates advanced deep learning methods for accurate object pose estimation. They showed that the proposed pose estimation model achieved better accuracy than common AR systems while satisfying real-time performance requirements.

Bastos et al. [73] presented a case study in which an augmented reality application was developed to optimize the workflow of a company. The application was used to aid electricians in performing maintenance operations through a neural network built with Tensorflow for recloser detection. Testers considered it a major asset for training newcomers, generalist electricians, and other workers who intend to maintain reclosers.

Li et al. [74] proposed an augmented reality-assisted deep learning-based approach to tackle three major challenges involving aviation connector inspection using a pretrained VGG-16 architecture.

Perez et al. [75] aimed to evaluate the application of CNNs for automated detection and localization of key building defects. The proposed model was based on pretrained VGG-16 and compared with the ResNet-50 and Inception models.

#### 5.3.2. Yolo—Deep Learning Network

YOLO (You Only Look Once) is a CNN architecture known for its remarkable performance and speed, making it advantageous for real-time AR systems. Malta et al. [76] used the YOLOv5 network architecture. A dataset was created with a total of 900 images, from which they identified eight classes. This model was quite fast, obtaining values of 0.012 s per image on test images, proving that it is a great choice for detecting objects in real time. The model was able to obtain accuracy above 96% and mAP(0.5:0.95) values above 79%. After testing the neural network, the same study proposed an architecture for a task assistance system to manage and process work orders on a CMMS. The work orders were sequences of procedures be performed by maintenance technicians through the indications provided by AR glasses.

Similarly, Zhi-Hao Chenÿ presented an automatic mechanism based on YOLOv4 to recognize potential cracks during nondestructive testing (NDT) of civic aeroengines. In their paper [77], training and testing images of task datasets were taken from the archives of the civic aircraft fuselage and engine repair records, theb various defects withn this dataset were categorized. A simple experiment compared different Faster R-CNN (Region-Based Convolutional Neural Network) and YOLOv4 framework models. Although the mean average precision (mAP) results were slightly higher for other networks, YOLO’s accuracy rate was slightly higher than Fast R-CNN, and Fast R-CNN’s loss score was higher than that of Faster R-CNN. Overall, the average times of the one-stage methods were much shorter than the two-stage methods. YOLOv4 was superior to the fastest detectors in terms of speed and accuracy. In tests performed with YOLOv4, the authors managed to obtain mAP values of 0.722 and detections of 0.0326 s. They concluded that these results indicate that the proposed system could considerably improve the accuracy and efficiency of this type of maintenance.

In order to create a more accurate object detection model for Augmented Reality using the communication between deep learning processing and the Microsoft HoloLens, ref. [78] proposed a study using several YOLO network models. The communication between the network and the glasses was organized as a client/server interaction through a local network capable of transferring both video streaming and data resulting from the recognition process. In terms of hardware, two NVIDIA Quadro P4000-type GPUs were used. On the server side, a darknet library was used to run the YOLO algorithms. On the client side, the glasses were used to collect input data and send the data to the server. When the server receives the frame used to perform object detection, the YOLO network is launched to process the received frame. In this case, the results included the annotation, bounding box, and box color of each detected object, containing the probability of an object belonging to a certain class. The server sends these results to the user, who can see the resulting object detection through the HoloLen in full detail and in real-time. The study used three versions of YOLO: YOLOv1, YOLOv2, and YOLOv3. The results showed the efficiency of YOLO algorithms for the average accuracy of different objects in an office. YOLOv3 showed great accuracy of over 90% for the total objects detected. Likewise, the average accuracy was around 96%. YOLOv1 and YOLOv2 did not reach 80%. To conclude, this approach to detecting and recognizing objects through HoloLens shows excellent detection results. In [79], the authors described an AR system that enables inventory, information retrieval, and information update directly on site. Their approach used tiny YOLOv2 pretrained with the COCO dataset, and they obtained an mAP value of 62.81%.

#### 5.3.3. MobileNet—Deep Learning Network

Estrada et al. [10] designed a smartphone app to create a learning platform for teaching students how to use electrical lab equipment. They combined a MobileNet-SSD v2 neural network and AR technology. Using this network, the mean average precision of detection was 81.4%, while the average recall of the model was 85.3%. In addition, they developed a multimeter tutorial in which virtual models were superimposed on real multimeters. The Unity 3D game engine was used as the primary development tool for this tutorial, which integrated deep learning (DL) and AR frameworks to create immersive scenarios.

Another AR application was implemented in [80] to detect a breadboard and instruct students on how to build a circuit. The system scanned a circuit diagram for circuit symbols and their connections. These components were then arranged using MobileNet, providing an accuracy of more than 92%. Students can use this application as a guided tutorial to build real circuitry.

## 6. Advantages and Disadvantages of AR Using Deep Learning Algorithms

Despite the promising developments discussed above, there are a number of relevant disadvantages to using the combination of AR and deep learning technologies. While the many advances made are intended to simplify and improve workers’ daily lives, there are always additional points to be discussed and possibly improved. Among the potentially negative aspects are, for example, that very large diversity of objects in production environments, which may be difficult to identify and track in real time [81]. Noise may interfere with voice-controlled systems, while illumination or occlusion problems may make object recognition difficult. With respect to deep learning, data collection is time-consuming and resource-intensive. These and other problems are currently the subject of intensive ongoing research.

However, published works have demonstrated great benefits as well. Kyeong-Beom et al. [69,82] proposed a smart task assistance method combining object detection and instance segmentation with wearable AR technology to provide more effective visual guidance with less cognitive load. They conducted two user studies to evaluate practical tasks used in realistic manufacturing, such as object matching, inspection, and maintenance. The authors stated that the proposed method was able to effectively reduce cognitive load and provide better task performance. They implemented several applications based on the proposed AR approach that further verified its effectiveness.

Etonam et al. [83] provided an overview of the potential benefits of adopting AR systems in the manufacturing industry, including time savings, reduced costs, and fewer defective products.

Table 2 summarizes the studies discussed above and the results of previous research involving the use of neural networks and AR for maintenance tasks.

## 7. Discussion and Perspectives

Augmented reality is a technology that has had and continues to have limitations. Current limitations include tracking techniques [84], which are often computer intensive and not 100% accurate, as well as the use of HMDs or other portable displays. While HMDs provide a more realistic experience, they are a heavy weight that needs to be carried on the head [85]. The level of interaction and user interfaces are additional problems that need to be dealt with. Interaction with AR devices is dependent on certain gestures and sounds, which can be easily mistaken in industrial environments. Another problem is that computer vision problems such as object location and identification require more efficient approaches. As noted by recent studies of the state of the art in deep learning, the greatly improved results in terms of generality and accuracy come at a cost in terms of the high performance required for computation. Improved performance is needed to find small objects or to enable operation in highly complex environments [86,87]. AR is a technology that has the ability to be used in conjunction with other technologies, and its combination with deep learning represents one of the most powerful and successful approaches to object detection and classification, greatly enhancing AR applications and experiences.

Table 2 lists a number of applications that combine AR and deep learning to achieve better maintenance results. The positive impact of using augmented reality technology to improve the results of maintenance assistance applications can be seen. DL and AR provide real-time, interactive, user-centered, and user-friendly intelligent applications, with a focus on enhancing the quality of experience and quality of service for end users [88].

Several studies, such as [89], have discussed recent advances in the Internet of Things (IoT). The large volume of data generated by IoT devices requires further intelligent data analysis and processing methods, including deep learning. In [90], Nosratabadi et al. carried out a review of the state of the art in deep learning and machine learning for smart cities. E-health is another growing area [91], among many others.

Through object detection, image processing, and computer vision, it is possible to enhance and improve AR efficiency and effectiveness and to develop more interactive and intelligent applications. In [92], Englert et al. cited several recent studies in which CNNs were utilized to perform computer vision tasks which could be applied in AR scenarios.

A final issue is that there are issues with cost-effectiveness compared to traditional methods. The development of an AR system involves choice of hardware as well as the development of software, which usually involves custom algorithms and data flows. These selections are challenging due to the wide variety of available services and options, which results in fragmentation, leading to different development processes and different user experiences [93]. However, the future prospects for the use of AR in learning, training, and optimization of industrial jobs, as well as in other areas, are very high. The new technologies and methods have immense potential, and there is growing interest in AR and its potential applications, as highlighted by Table 2 including four articles from 2019, six from 2020, three from 2021, and two from 2022.

## 8. Conclusions and Future Work

Augmented reality has become one of the most promising technologies in maintenance thanks to the ease with which it can connect the physical environment to virtual content.

We analyzed a number of articles proposing diverse scenarios applying AR to assist in maintenance tasks. From our review, it is clear that this technology can help technicians, engineers, operators, etc., in varied maintenance tasks, including learning, assistance, remote support, inspection, and more. The potential of this technology increases even more when combined with deep learning techniques for object detection, which is the primary focus of this paper. It is necessary to bear in mind that for the practical use of these systems it is necessary to carry out environmental and personal studies and analyses in order to optimize their use as much as possible.

In addition to the proposals described herein, there are many others in the same area of study that are equally important in understanding the future of augmented reality and neural network training for the identification and location of objects.

This article has provided contributions in several areas. We have reviewed state-of-the-art machine learning techniques in combination with new and existing augmented reality applications to to assist maintenance technicians while increasing safety and efficiency.

Our future work will includes further literature research on object occlusion and augmented reality hardware.

## Figures and Tables

**Table 1:** Summary of the uses of augmented reality in maintenance tasks.

Problem	Hardware	Results
Maintenance tasks inside an armored vehicle	HWD: 1280 × 1024 px and 60${}^{\circ }$ diagonal FOV; HWD: 800 × 600 px and 34${}^{\circ }$ diagonal FOV	Average task completion: 42 s; Average task location: 4.9 s.
Combustion chamber assembly and disassembly process	NVIS nVisor ST60: 1280 × 1024 px 60${}^{\circ }$ diagonal FOV	Task execution was 21.31 s or 46.8% faster using AR.
AR instructions for LEGO buildings	Sony Glasstron LDI-100B 30${}^{\circ }$ diagonal FOV	The system can improve task performance (lower average time and number of errors).
AR-based real-time remote maintenance support	Microsoft HoloLens	Minimizes the MTTR.
AR systems with a CMMS to plan for preventive and predictive actions	Microsoft HoloLens	The system can minimize unexpected failures.
AR system for maintenance and repair of an electrical system	-	The system can reduce maintenance cost and reduce human error.
AR system to improve operation and training for power grid operations staff	-	Enhanced training and effectiveness,
AR application to facilitate the use of a voltage control panel	-	Reduction in intervention preparation time and increased level of safety.

**Table 2:** Summary of the uses of neural networks and augmented reality in maintenance contexts.

Network	Some Results	Hardware	Problem
CNN	Disassembly task was 60 min	Microsoft HoloLens PC—Intel i7, 16 GB of RAM and 8 GB of GPU	Assembly and disassembly of combustion chambers [65]
CNN	Improved maintenance efficiency and effectiveness.	Microsoft HoloLens	Remote control tower maintenance and induction system [66]
YOLOv1	System mAP: 71.44 and FPS: 4.2	Microsoft HoloLens 2 NVIDIA Quadro P4000-type GPU	Recognize Objects [78]
YOLOv2	System mAP: 79.74 and FPS: 4.6		
YOLOv3	System mAP: 96.28 and FPS: 5		
YOLOv4	System mAP: 72.2 and FPS: 30.6	Conventional GPU NVIDIA Jetson TX2	Recognize potential cracks in aeroegine [77]
YOLOv5	System mAP: 79.7 and FPS: 83.3	Tesla v100	Recognize car engine parts [76]
MobileNet V2	Testing images: mAP: 0.81 Samsung FPS: 8.5 One Plus FPS: 5.5	Android plataforms	Platform for teaching how to use electrical equipment [10]
MobileNet	Dataset with 15 classes Training mAP: 92.0	Android plataforms	AR visualization of the expected circuit on a breadboard [80]
Mask R-CNN ResNet101	-	Microsoft HoloLens NVIDIA GeForce GTX 1080 Ti (11 GB)	Task assistance [69,82]
Faster R-CNN	Detection in five real tools: mAP: 84.7	AR-display NVIDIA GTX 1080 Ti GPU	Mechanical assembly instruction [67]
VGG-8	Dataset: 7290 samples Proposed approach: mAP: 85.69	Wearable AR device	Manage aircraft cable assembly process [68]
SSD MobileNet	Task: Damage detection mAP in 120 test images: 91.67	Epson BT-300	Enabling automated damage detection in real-time [70]
Encoder–decoder CNN-ResNet18	Proposed pose estimation model achieved better accuracy than competing methods	Microsoft HoloLens Nvidia Titan X GPU (12 GB)	Improving IoT-AR by integrating DL with AR [72]
Neural Network	-	Realwear HMT-1	Optimizing workflow [73]
Combination of FPN and BiLSTM	Tests in pin detection mAP: 99.00	AR glasses and two GPUs (RTX 2080)	Three tasks in aviation connector inspection [74]
Based on pre-trained VGG-16	Test overall accuracy: 87.50	-	Detection and localization of key building defects [75]

## Data Availability

Not applicable.

## Abbreviations

The following abbreviations are used in this manuscript:

API	Application Programming Interface
AR	Augmented Reality
ARMAR	Augmented Reality for Maintenance And Repair
BMW	Bayerische Motoren Werk
CAD	Computer-Aided Design
CMMS	Computerized Maintenance Management
CNN	Convolutional Neural Network
COCO	Common Objects In Context
CPU	Central Processing Unit
CV	Computer Vision
DL	Deep Learning
FOV	Field Of View
GPS	Global Positioning System
GPU	Graphics Processing Units
HMD	Head Mounted Device
HUD	Head-Up Display
HWD	Head-Worn Display
IoT	Internet of Things
KARMA	Knowledge-based Augmented Reality for Maintenance Assistance
LCD	Liquid Crystal Display
MAP	Mean Average Precision
MR	Mixed Reality
MRTK	Mixed Reality Tool Kit
MTTR	Mean Time To Repair
NASA	National Aeronautics and Space Administration
PC	Personal Computer
QR	Quick Respons
R-CNN	Region-Based Convolutional Neural Networks
SLR	Systematic Literature Review
USMC	United States Marine Corps
VR	Virtual Reality
YOLO	You Only Look Once
